# Accumulation of production capital and income growth of Chinese farmers in the post-poverty alleviation era: A study based on a two-way fixed effects model with CFPS data

**DOI:** 10.1371/journal.pone.0309723

**Published:** 2024-09-13

**Authors:** Zhao Liu

**Affiliations:** Business School, Henan Mechanical and Electrical Vocational College, Zhengzhou, China; Huanggang Normal University, CHINA

## Abstract

Agricultural mechanization is a crucial indicator of modernization in agriculture. It improves productivity and underpins the evolution of a modern state. This study scrutinizes the enduring effects of government subsidies on farm machinery acquisition, income growth, and capital accumulation in rural households. It is based on policies about targeted poverty alleviation and rural revitalization. Research findings indicate that government subsidies have significantly increased the per capita net income of rural households. However, in the post-poverty alleviation era, for households that already possess agricultural machinery, the benefits brought by government subsidies in the early stages of the policy cycle tend to diminish over time. From 2016 to 2020, government subsidies continued to enhance the value of agricultural machinery in rural households. Their impact on ownership rates first slightly declined and then increased again. The promotional effect in 2020 was not significantly better than in 2016. When China is fighting against poverty, it is essential to encourage rural households to use their income and government subsidies to accumulate production capital. A long-term mechanism has been established to protect the achievements of poverty alleviation, promote agricultural mechanization and rural modernization, and support rural revitalization.

## 1. Introduction

In most developing countries, agriculture plays a significant role in the national economy and is a primary source of national wealth. Enhancing agricultural productivity is fundamental for advancing national development. Although agricultural mechanization is not the sole agricultural technology, it is the most visible technological change in rural areas of developing countries. Following the implementation of *Chinas Agricultural Mechanization Promotion Law* (2004) and its repeated emphasis in the *Central No*.*1 Document*, China’s agricultural machinery industry substantial growth. For farmers, higher levels of agricultural mechanization lead to improved productivity and increased income.

After achieving the historic milestone of complete poverty alleviation in 2020, the income trends for Chinese farmers have generally been positive. However, despite this rise, the growth rate of farmer incomes still lags the overall national economic growth. Under the current rural revitalization policy context, Chinas rural areas are at a critical juncture, transitioning from traditional farming methods to agricultural modernization. Preventing a return to poverty and maintaining income growth for farmers have become crucial issues.

**Fig 1:**
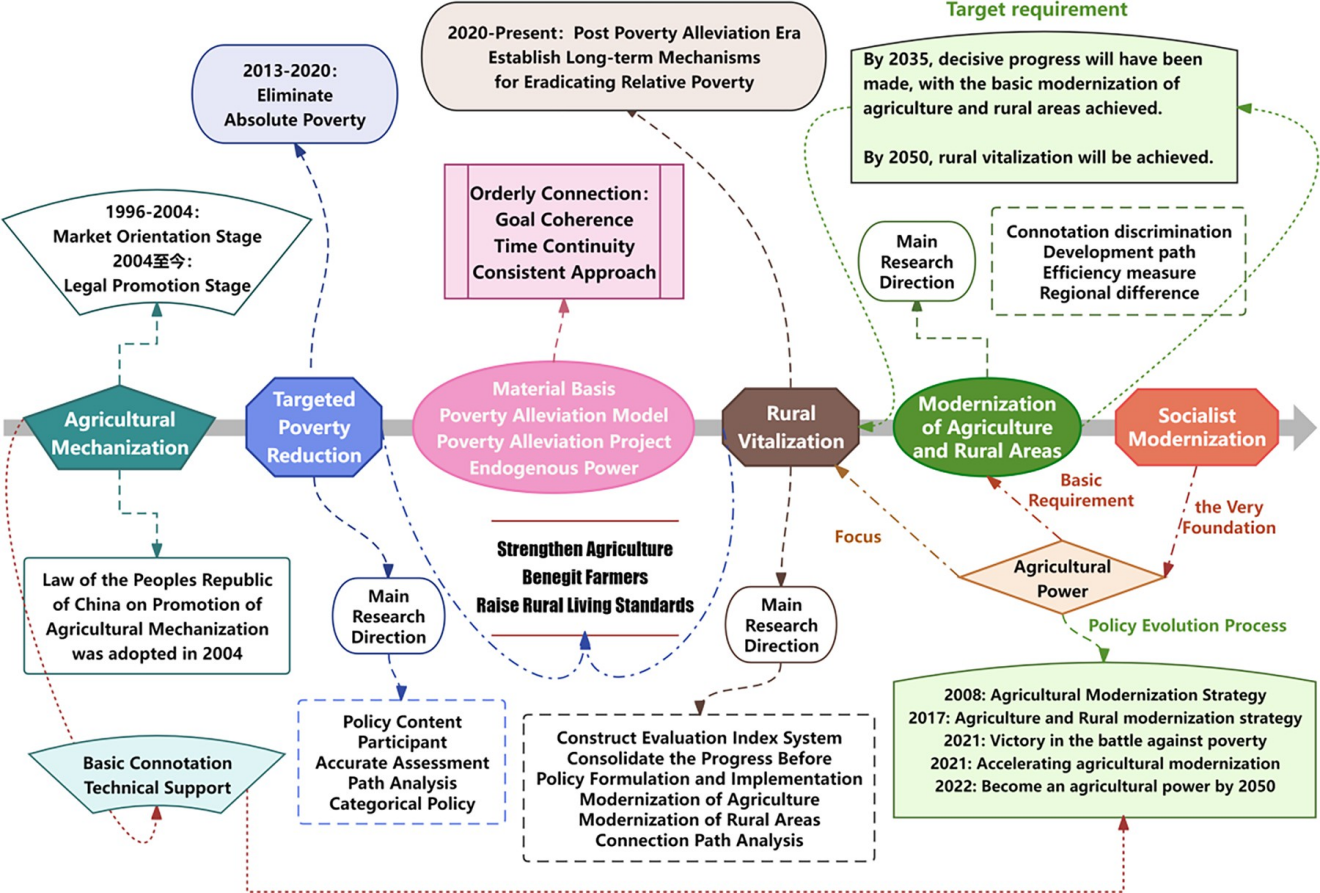
Framework of policy analysis.

Fig 1 illustrates the development process of agricultural mechanization and the modernization of agriculture and rural areas in China, highlighting the research focus of scholars during the implementation of targeted poverty alleviation and rural revitalization strategies. Currently, China’s 14th Five-Year Plan and the *Opinions of the Central Committee of the Communist Party of China and the State Council on Fully Promoting Key Rural Revitalization Tasks in 2023* emphasize the importance of research and promotion of agricultural machinery. These efforts aim to advance key rural revitalization tasks by 2023 and achieve the strategic goal of "basically achieving agricultural and rural modernization" by 2035 [1–3].

However, due to limitations in the availability of agricultural subsidy data in China, research has often relied on theoretical analysis. When analyzing the income effects of agricultural mechanization, scholars have primarily focused on the income increase effects for households that already own agricultural machinery and the average impact on farmer income [4, 5]. There is insufficient research on the transformation of production capital among households and the sustained impact of policies.

When focusing on China in the research context, attention should be paid to the following three issues. Firstly, in terms of policy analysis, more scholars take enterprises as the main research object, exploring the impact of government subsidies on enterprise operations and performance. Currently, there are few scholars focusing on households as the family unit, with their research mainly targeting specific government subsidy policies, such as the subsistence allowance policy and the policy of returning farmland to forests [6]. Discussions on the overall implementation effects of government subsidy policies are not common. However, in reality, national development policies are typically implemented comprehensively in combination over a relatively long period of time. When discussing the effects of individual subsidies, it is difficult to avoid the interplay among different subsidies. At the same time, a small number of scholars still believe that government assistance does not significantly reduce poverty [7, 8]. Secondly, in terms of poverty types, many scholars focus on absolute poverty. However, for China, absolute poverty was eliminated in 2020. With the acceleration of urbanization and the widening urban-rural income gap, preventing a return to poverty has become a new challenge while further addressing relative poverty. Thirdly, many scholars have noticed the impact of government subsidies on household income, but agricultural machinery is the core production tool for rural households. Therefore, this study pays more attention to whether this part of the income can be transformed into production capital and create wealth in the future, forming a mechanism that effectively prevents a return to poverty in the long term.

In response, this paper employs a difference-in-differences method and analyzes CFPS data from 2014–2020 using a two-way fixed effects model to evaluate the long-term effectiveness of government subsidies in continuously promoting income growth and agricultural mechanization among farmers. This study aims to fill the gap in this research field, providing a reference for the formulation of complementary policies for a group of households in China who do not yet own agricultural machinery but may see significant changes with policy assistance in the future. Additionally, this research aims to provide empirical data for other developing countries as well.

## 2. Theoretical mechanism and research hypotheses

### 2.1 The relationship between government subsidies and income growth of rural households

Keynes posited that government expenditure can stimulate aggregate demand via the multiplier effect, thereby fostering economic growth. Through direct subsidies and fiscal support such as social security and agricultural subsidies, the government transfers funds to rural households, significantly boosting farmers’ incomes. These funds can be used for consumption, investment, and improving living conditions, as well as for savings or investment in production capital. Accumulation of production capital refers to the increase in material and human capital used for producing goods and services. For rural households, this includes land improvement, purchasing agricultural machinery and equipment, investing in high-quality seeds, fertilizers, and pesticides, among others. Although rural areas often suffer from inadequate production capital and technological backwardness, leading to low agricultural production efficiency and slow household income growth, rural households receiving additional capital injections, such as government subsidies, can accumulate production capital through direct capital investment (purchasing production materials like seeds, fertilizers, and agricultural machinery) and technological upgrades (participating in training programs to enhance production techniques and knowledge). This helps improve production efficiency, expand output, and ultimately achieve income growth. Additionally, subsidies for returning farmland to forests encourage farmers to engage in non-agricultural industries in their spare time, thereby increasing overall income levels.

International experience indicates that effective subsidy policies are crucial for promoting sustainable agricultural mechanization development and increasing farmers’ incomes. Government agricultural subsidy policies originated in the United States in the 1930s, but formal research on agricultural subsidies began with the establishment of the European Community in 1957 and the implementation of its Common Agricultural Policy. Audsley E and B. Recio pointed out that there is a positive correlation between government direct subsidies and farmers’ total income: the more subsidies the government provides to local governments, the higher the direct total economic benefits for farmers [9, 10]. Over decades, developed countries’ agricultural subsidy policies have shifted from primarily supporting prices to mainly supporting incomes, reducing subsidies for agricultural product prices and import-export activities, and moving towards balanced subsidies for agricultural products and production factors, with greater emphasis on ecological support and environmental protection. Currently, international research on agricultural subsidy policies primarily involves theoretical foundations, subsidy levels, policy analysis, and their impact on stakeholders. Despite varying circumstances across countries, many developed nations continue to implement various subsidy policies to promote agricultural mechanization even after achieving initial success in agricultural modernization [11]. Despite differences in subsidy methods and structures, the absolute amount of subsidies generally exhibits an upward trend. These findings provide practical insights and scientific references for developing countries like China. In practice, local governments in China follow central policies and draw on the advanced experiences of developed countries, introducing various subsidy strategies to sustain this trend. Chinese scholars generally agree that government subsidies indeed increase farmers’ incomes. However, some argue that although agricultural subsidy policies have been implemented in China, their effects are not entirely satisfactory [12]. Therefore, it is necessary to study the practical experiences and lessons of these policies and propose feasible recommendations for sustainable and healthy agricultural development. Considering this, Hypothesis 1 is proposed:

**Hypothesis 1 (H1):** Under the context of rural revitalization, the implementation of government subsidies positively promotes income growth among rural households in China.

### 2.2 The relationship between government subsidies and the value of agricultural machinery owned by rural households

According to the Solow growth model, from the perspective of production capital, government subsidies can provide additional financial support to help rural households purchase or invest in more agricultural machinery and equipment, improve the quality and quantity of production tools, enhance productivity and output, thereby increasing the value of agricultural machinery owned by the household. From the standpoint of technological progress, government subsidies can also be used for participating in agricultural technology training, assisting farmers in better utilizing and maintaining agricultural machinery and equipment, further improving production efficiency, and maximizing the utility of the machinery. With capital accumulation and technological progress, agricultural production may experience economies of scale. From the investment and savings perspective, government subsidies can increase household disposable income, with some income able to be converted into savings for future capital reinvestment or purchasing more and higher-value agricultural machinery.

Research on agricultural machinery began in the last century among international scholars, and Western developed countries have largely achieved agricultural mechanization, resulting in relatively mature studies. However, due to significant differences in household registration systems between the West and China, there is less research focusing on the value of machinery and household income in China. Scholars generally agree that the rapid development of agricultural mechanization can enhance farmers’ income. Extensive empirical research indicates that government subsidies, by promoting the upgrade of agricultural machinery, can reduce agricultural costs, improve production efficiency, optimize agricultural structure, and thereby increase farmers’ incomes. Ngxetwane found that increasing machinery value can raise agricultural yields and reduce production costs, thus raising farmers’ incomes [13].

As China’s economic and technological levels improve, the pace of agricultural mechanization development has accelerated, leading to increased research by Chinese scholars on related issues. Chen Yan argued that the accelerated process of agricultural mechanization in China helps achieve agricultural and rural modernization within the context of rural revitalization, thereby promoting farmers’ income growth [14]. Yu Wuran suggested that enhancing the agricultural machinery industry and the level of agricultural mechanization, and comprehensively improving agricultural production capacity, contributes to China’s sustainable agricultural development, promotes farmers’ income growth, and advances rural revitalization [15]. Based on this, the second hypothesis is proposed:

**Hypothesis 2 (H2):** In the context of rural revitalization, the implementation of government subsidies positively promotes the enhancement of agricultural machinery value among rural households.

## 3. Research design

### 3.1 Indicator selection

Chinese agricultural operations are typically decentralized, with individual households as the primary operational units, differing from the large-scale farms common in developed agricultural nations. This structure leads to lower levels of mechanization, with most agricultural labor being manual or semi-automated. To align with China’s national conditions, this study utilizes data from the China Family Panel Studies (CFPS) database. According to research by Kong Fanjin, farm machinery constitutes the primary capital for future production for most households with rural registration [16]. Key strategies for increasing household income include expanding the scope of machinery purchase subsidies, broadening the types of machinery covered by policies, and promptly updating the subsidy catalog to include new machinery types. These measures can improve the efficiency of government subsidy funds, promote the accumulation of productive capital and enhance the level of agricultural mechanization. Consequently, this study selects the value of agricultural machinery from CFPS as the primary metric for measuring productive capital.

In evaluating household income, many scholars prefer to use per capita income, particularly per capita disposable income [17, 18]. Per capita disposable income focuses on the actual residual funds after daily consumption and savings, making it a more suitable measure for assessing the financial status of households post-purchase of farm machinery and their ability to maintain and utilize it. However, calculating per capita disposable income can be complex, potentially introducing calculation errors or data collection difficulties, and it can also be influenced by uncontrolled variables, ultimately affecting the accuracy of the assessment. Theoretically, funds for agricultural mechanization investment primarily come from household net income, so the level of net income directly affects investment in agricultural mechanization and has a certain lag effect meaning the net income of the previous year positively impacts the agricultural mechanization investment of the following year. However, the per capita net income indicator primarily reflects a household’s basic economic strength and its capacity for investment in agricultural production. Although using per capita net income may overestimate the actual disposable funds of a household, it provides a clear understanding of the direct effect of government subsidies on net income. Therefore, this study selects household per capita net income to measure the economic status of rural households, with data adjusted to 2010 constant prices. According to the China Family Panel Studies (CFPS), with 2010 as the survey base year, the households and individual family members surveyed that year will be tracked as permanent subjects for follow-up surveys, with a cross-round tracking rate of over 85%. Surveys are conducted every two years.

To address potential endogeneity issues due to omitted variables, this study incorporates household size, healthcare expenditure, and education and entertainment expenditure as control variables to account for factors such as household member health and children’s education [19].

According to Becker’s theory of household production, households are not only consumption units but also production units. Household size is closely related to household resource allocation and utility maximization. A larger household size means that total income needs to be divided among more family members, resulting in a decrease in per capita income. It also implies that with limited resources, households will prioritize basic life needs, thus reducing spending on advanced production capital such as agricultural machinery.

According to Grossman’s health capital model, health itself is a form of capital, and household expenditures on healthcare represent investments in health capital. High healthcare expenditures mean that a large portion of disposable income is allocated to health expenses, reducing funds available for other economic activities and leading to an overall decline in economic conditions. Furthermore, a decrease in household investments in production capital such as agricultural machinery will hinder farmers from acquiring new machinery or upgrading existing equipment.

Based on the household budget constraint model, household income is allocated among different categories of expenditure such as food, housing, healthcare, education, and entertainment to maximize the total utility of household members. When expenditures on entertainment and education are high, the funds available for savings and investment are reduced, affecting the accumulation of pure income. Simultaneously, a decrease in funds available for purchasing and maintaining agricultural machinery will also impact the appreciation of machinery value.

Regarding government subsidies, detailed data on subsistence allowances, subsidies for converting farmland to forests, agricultural subsidies, five-guarantees subsidies, and poverty subsidies have not been recorded separately in the CFPS since 2016. Instead, they are aggregated under a single category of government subsidies. To maintain consistency in data standards, this study aggregates data for various government subsidies before 2016. Additionally, whether a household receives government subsidies is used as a control variable, with a value of 1 if the household receives subsidies and 0 otherwise.

### 3.2 Data source and descriptive statistics

The data for this study are drawn from the CFPS mixed panel data from the years 2014, 2016, 2018, and 2020, covering 31 provinces in China. During sample selection, samples with urban household registration, missing key indicators, and distorted data were excluded. Logarithmic transformations were applied to some data to reduce errors. The descriptive statistics of the main variables are shown in Table 1.

**Table 1:** Descriptive statistics of variables.

Variable Type	Variable Name	Subsidy Status	2014	2016	2018	2020
Dependent	Per capita household net income (RMB)	With subsidy	8959.25	9124.44	11541.45	17170.00
Dependent	Per capita household net income (RMB)	Without subsidy	13335.05	13912.61	16148.14	20910.00
Independent	Agricultural machinery ownership rate (%)	Without subsidy	0.2127	0.2775	0.2468	0.258
Independent	Agricultural machinery ownership rate (%)	With subsidy	0.4866	0.4618	0.4082	0.4351
Independent	Agricultural machinery value (RMB)	Without subsidy	1418.53	1974.41	1780.11	2160.49
Independent	Agricultural machinery value (RMB)	With subsidy	2219.98	3292.87	3821.25	4484.74
Control	Family size (person)	-	4.01	3.98	3.86	3.97
Control	Government subsidy (%)	-	0.677	0.586	0.5813	0.4922
Control	Culture, education and entertainment expenditure (RMB)	-	3356.11	3824.26	4530.48	4542.28
Control	Healthcare expenditure (RMB)	-	4633.72	5292.5	5510.21	5222.53

Based on the aforementioned statistics, notable disparities emerge in both the value and ownership rate of agricultural machinery between households that receive subsidies and those that do not. Among non-subsidized households, the ownership rate of agricultural machinery experienced a significant increase in 2016, a slight decline in 2018, followed by another risein 2020, albeit failing to surpass the level two years prior. Conversely, subsidized households witnessed a decrease in machinery ownership from 2014 to 2018, with a subsequent upturn in 2020. This trend prompts further scrutiny. The persistently low ownership rate of machinery among non-subsidized households may stem from their reduced involvement in agricultural production, leading to an average machinery value notably lower than that of subsidized households. The adoption of targeted poverty alleviation strategies led to a marked decrease in the proportion of households receiving government subsidies in 2016, with stabilization in 2018 and further decline by 2020, signifying a shift in government subsidy policies from a "broad-based" to a "targeted" approach.

Regarding key metrics such as per capita net household income and household agricultural machinery ownership, the study distinguishes between subsidized and non-subsidized households. Overall, households receiving subsidiesdemonstrated robust growth in per capita net income, while non-subsidized households exhibited a comparatively slower yet consistent upward trajectory. Specifically, from 2014 to 2016, both groups saw a muted change in per capita net household income. However, in 2018 and 2020, significant increases were observed, with incomes for subsidized and non-subsidized households rising by 26%, 16%, and 49%, 29% respectively over the two years. The income disparity between the two groups initially widened from 4376 in the initial 2016 statistics to 4788, followed by a continuous decline to 4607 and 3740. This underscores the enduring efficacy of government subsidy policies in bolstering rural household incomes and mitigating income inequalities over the long term.

Household expenditures on healthcare, education and entertainment showed consistent growth from 2014 to 2018, indicative of improved living standards. However, in 2020, despite the rise in per capita income, these expenditures witnessed a decrease, possibly attributable to the impact of the COVID-19 pandemic on travel and medical services.

## 4. Empirical analysis

### 4.1 Model setup

Prior to estimating the model, this paper ensures the rationality of the model setup through the following steps: first, examining the correlation between government subsidies and each control variable with farmers’ incomes; second, assessing the presence of multicollinearity between government subsidies and various control variables; finally, determining whether a two-way fixed effects model should be employed for the estimation.

The results of the correlation levels between variables are presented in Table 2. The first column displays the correlation between each explanatory variable and per capita net income. The findings reveal that the correlation coefficient between per capita net income and the value of household agricultural machinery is 0.183, significant at the 1% level, suggesting a noteworthy positive correlation between them. Similarly, other control variables also exhibit significant positive correlations with per capita net income, each at the 1% significance level. Thus, the inclusion of the explanatory variables in this model is deemed reasonable.

**Table 2:** Correlation test of variables.

	lny	Agricultural machinery ownership rate	Agricultural machinery value	Family size	Health care expenditure	Education and entertainment expenditure
lny	1					
Agricultural machinery ownership rate	0.196***	1				
Agricultural machinery value	0.183***	0.673***	1			
Education and entertainment expenditure	0.852***	-0.256***	0.067*	1		
Family size	0.132***	0.796***	0.728***	-0.296***	1	
Health care expenditure	0.693***	0.123**	0.156**	0.323***	0.034	1

Note

* p <0.1

* * p <0.05, and

* * * p <0.01.

Due to the strong logical connections among the various explanatory variables, there may be issues with multicollinearity. Therefore, a multicollinearity test was performed on the dependent variables. As seen in Table 3, the variance inflation factor (VIF) for each variable is below 10. This indicates that there is no severe multicollinearity among the explanatory variables, ensuring the accuracy of the estimation results.

**Table 3:** Multicollinearity test.

Variable	Agricultural Machinery value	Agricultural machinery ownership rate	Family size	Health care expenditure	Education and entertainment expenditure
VIF	6.230	5.186	2.856	1.697	1.346

Since changes in household characteristics and time factors can affect per capita net income, this paper conducts a Hausman test to eliminate potential omitted variable bias from specific poverty alleviation policies or unobservable changes in household characteristics in certain impoverished areas. Table 4 shows that the P-value is less than 0.01, rejecting the null hypothesis of using random effects. Additionally, a Wald test is performed to examine for individual time effects. The results indicate that the P-value is less than 0.01, strongly rejecting the null hypothesis of "no time effects".

**Table 4:** Hausman test.

	Sargan-Hansen statistic	Chi-sq(6)	P-value
Agricultural Machinery Value	14.783	13.102	0.006
Agricultural Machinery Ownership Rate	17.146	11.987	0.008

The difference-in-differences (DID) method, a crucial tool for policy evaluation, has been widely used in international economics since 1978 and was adopted by Chinese scholars, starting from Zhou Li’an et al. to assess the effectiveness of major policy reforms [20–22]. The DID method is based on the premise of a natural experiment, viewing the policy as an exogenous event independent of the economic system. It estimates the net effects of the policy by comparing dynamic both longitudinally and cross-sectionally. In regression models, the samples affected by the policy constitute the treatment group, while those unaffected form the control group. By comparing the changes before and after the policy implementation between these two groups, the net effect of the policy is determined. Therefore, this paper views the government subsidy policy implemented after the targeted poverty alleviation strategy in China in 2014 as a "quasi-natural experiment." Households receiving government subsidies post-2014 make up the experimental group, while the rest form the control group. The paper applies DID method and uses a two-way fixed effects model to control for other potential macroeconomic factors, aiming to achieve more reliable estimation results. The baseline model (1) is set as follows:

$$\ln\;y_{it}=\beta_{0}+\gamma (Treat_{\mathrm{it}}\times Y{\mathrm{ear}}_{it})+\theta Treat_{it}+\rho Year_{\mathrm{it}}+\lambda X_{it}+\varepsilon_{it}$$ (1)

*β*_0_ is the intercepted item. ln *y*_*it*_ represents the natural logarithm of the per capita net income of family i at time t. *Treat*_it_ is the binary variable indicating whether family i is part of the treatment group at time t, receiving the government subsidies (1) or not (0). *Y*ear_*it*_ is a binary variable indicating the post-policy period for all families at time t, with 0 for 2014 1 for subsequent years. *T*reat_*it*_×*Y*ear_*it*_ is the interaction term representing the effect of the treatment in the post-policy period. The estimated DID coefficient, *γ*, captures the causal impact of government subsidies on the per capita net income of rural households by comparing the treatment group to control group in the post-policy period. *X*_*it*_ represents the other control variables. *familysize*_*it*_ is the logarithm of family size, med_*it*_ represents health care spending. *eec*_*it*_ represents culture and recreation spending. Since the large number of sample data in the latter two indicators is 0, here, we use the first value plus 1, then take the logarithmic processing method. *ρ* and *λ* is the corresponding coefficient. *T*_t_ is the time-specific fixed effect capturing common shocks or trends at time t affecting all families. *ID*_*i*_ is the family-specific fixed effect capturing unobserved, time-invariant characteristics of family i. *ε*_it_ is the error term. Based on model (1), the DID model (2) is set as follows:

$$\ln\;y_{it}=\beta_{0}+\gamma (Treat_{\mathrm{it}}\times Y{\mathrm{ear}}_{it})+T_{t}+ID_{i}+\lambda X_{it}+\varepsilon_{it}$$ (2)

The sample data are divided into three groups: the full sample group, the sample group with agricultural machinery, and the group without agricultural machinery. The analysis investigates the impact of government subsidies within the framework of targeted poverty alleviation and rural revitalization policies on farmers ownership of agricultural machinery. Due to space constraints, the results for control variables such as family size, healthcare expenditure, and education and entertainment expenditure (in logs) are not included.

### 4.2 Empirical results analysis

Table 5 presents the main regression results. The findings indicate that in 2016, during the initial phase of government subsidies, there was a significant positive effect on the per capita net income of recipient households. The coefficient of the interaction term between the time dummy variable and government subsidies is 0.136, suggesting that the per capita net income of subsidized households in 2016 was 13.6% higher than in 2014, significant at the 5% level. However, by 2018, this coefficient had declined to -0.03 and was no longer significant. These results can be attributed to two primary reasons:

**Table 5:** Impact of government subsidies on household net income.

Variable	Full sample (1)	With Agricultural Machinery (2)	Without Agricultural Machinery (3)
treat_16	0.136**	0.156**	0.060***
	(0.04)	(0.07)	(0.05)
treat_18	-0.030	0.066	-0.064
	(0.04)	(0.07)	(0.05)
treat_20	0.170***	0.004	0.171***
	(0.04)	(0.07)	(0.05)
Familysize 16	-0.028***	0.019	-0.023*
	(0.01)	(0.02)	(0.01)
Lnmed	0.028*	0.016*	0.028*
	(0.00)	(0.01)	(0.01)
Lneec	0.011***	0.003	0.009*
	(0.00)	(0.01)	(0.00)
_cons	8.764***	8.539***	8.804***
	(0.04)	(0.09)	(0.06)
N	22899	7808	15091
r2	0.093	0.119	0.077

Note

* p <0.1

* * p <0.05, and

* * * p <0.01.

1. The diminishing marginal returns of government subsidy policies during the poverty alleviation period. Initially, the government significantly boosted the income of impoverished households by accurately allocating resources and facilitating the accumulation of productive capital. However, over time, the growth in rural household income hit bottlenecks. As the economic conditions of rural households improve nationwide, it becomes essential to thoroughly understand the underlying causes of poverty and develop more comprehensive and targeted poverty alleviation policies to effectively assist the remaining impoverished households.
2. 2017 was a transition year for China’s Stage III emission standards for tractors, which impacted the effectiveness the subsidy policy in wealth creation. Consumer hesitancy about the performance and prices of Stage III products led to delays or cancellations of new machine purchases. Furthermore, provinces such as Henan, Inner Mongolia, Jiangsu, and Anhui reduced tractor subsidy amounts during this period, increasing the cost of acquiring new machinery.

However, by 2020, the coefficient of the interaction term rebounded to 0.17 and was significant at the 1% level. Policy reviews indicate that 2018 marked the beginning of a new round of agricultural machinery purchase subsidy policies. Compared to previous years, these policies expanded the subsidy scope, supported tools for agricultural green development, and removed application thresholds for subsidies, thereby stabilizing farmers’ expectations for machinery purchases. As a result, government subsidies once again promoted income growth among rural households.

For the entire sample, the results of the regression analysis reveal that the coefficient for household size is -0.028, significantly negative at the 1% level. This implies that larger households have a more pronounced negative impact on per capita income, emphasizing the economic burden of elderly care and education [23]. Additionally, the coefficients for healthcare expenditure and education and entertainment expenditure are 0.028 and 0.011, respectively. Though small, they are statistically significant and positive, indicating that even with low income, expenditures on healthcare and education can potentially yield economic benefits. In other words, investing in healthcare and education can lead to indirect economic advantages, even in situations where if the income levels are not high.

After analyzing the average effects on the total sample, this paper further evaluates the heterogeneous impact of government subsidies on different households to identify the primary target groups for policy intervention. Comparing the regression results in the second and third columns, the findings indicate that government subsidies are particularly effective for households without agricultural machinery, especially in 2018 and 2020. In these years, the effect of government subsidies on household income is consistent in both the full sample and the group without agricultural machinery, with similar coefficient sizes. Conversely, for households already owning agricultural machinery, the subsidies are less impactful, significant only in 2016, and further weakened by 2020, with a coefficient of only 0.004. In conclusion, consistent with Hypothesis 1, the paper concludes that in the post-poverty alleviation era, government subsidies do not significantly enhance income for households already owning agricultural machinery, whereas they can achieve a long-term effective increase in per capita income for households without agricultural machinery.

### 4.3 Robustness test

In addition to government subsidy policies, other policies or random factors may affect the economic growth of rural households. To further test the robustness of the results and exclude the interference of other factors, this study follows the approach of Weng Yijing et al. by altering the implementation time of government subsidies for a counterfactual test [24]. Specifically, considering household differences and time factors, it assumes advancing the implementation year of the government subsidy policy by one or two years, controlling for each variable, and performing a regression based on model (2). If the interaction term of the core explanatory variable remains significantly positive, it suggests that the income growth difference likely stems from other policy changes or random factors.

Table 6 presents scenarios where the government subsidy policy implementation time is advanced by one year (column 1) and two years (column 2). The results show that although the core explanatory variable interaction term is positive, it is not statistically significant. This indicates that the implementation of the government subsidy policy does indeed promote household economic growth, thereby validating the robustness of the DID analysis results.

**Table 6:** Robustness test results.

	(1)	(2)
*P*re_1_*Treat*_it_×*Year*_it_	0.065 (0.037)	
*P*re_2_*Treat*_it_×*Year*_it_		0.048 (0.029)
*Year*_it_	0.079* (0.049)	-0.019 (0.081)
_c*ons*	0.532 (0.692)	-0.398 (0.299)
*R* ^2^	0.592	0.635

## 5. Policy sustainability analysis

To evaluate the sustainability of government subsidies, this study uses the value of household agricultural machinery as an indicator of production capital accumulation. The model form is similar to Eq (1), but the dependent variable is the value of household agricultural machinery, measured in thousands of yuan. Additionally, a dummy variable indicating whether the household owns agricultural machinery is introduced. This variable takes a value of 1 if the value of agricultural machinery is greater than 0, and 0 otherwise. It serves as the dependent variable in the Probit model, which examines the impact of government subsidies on the ownership rate of agricultural machinery. At this stage, the control variable is limited to household size, since healthcare and education/entertainment expenditures lack sufficient theoretical support to influence the decision to purchase agricultural machinary. The study continues with a difference-in-differences model test, using the value of agricultural machinery and whether the household owns agricultural machinery as dependent variables. The results are presented in Table 7.

**Table 7:** Impact of government subsidies on household agricultural machinery status.

	Full sample	With agricultural machinery	Probit model
	Lnagr 1	Lnagr 2	New treat
treat_16	0.465***	-0.057	0.415***
	(0.10)	(0.09)	(0.04)
treat_18	0.531***	0.176**	0.372***
	(0.10)	(0.10)	(0.04)
treat_20	0.585***	-0.333***	0.396***
	(0.12)	(0.09)	(0.04)
familysize16	0.236***	0.027	0.135***
	(0.03)	(0.03)	(0.01)
_cons	1.673***	6.907***	-1.095***
	-0.132	-0.135	-0.031
N	22899	7808	22899
r2	0.026	0.085	

Note

* p <0.1

* * p <0.05, and

* * * p <0.01.

Based on the data in the first column, from 2016 to 2020, government subsidies had a statistically significant positive impact on the value of agricultural machinery, with this effect increasing over time. Following the implementation of the targeted poverty alleviation strategy, these subsidies effectively enhanced the value of agricultural machinery in rural households, continuously promoting the accumulation of production capital. The data in the second column shows that two years after the subsidy policy was implemented, there was no significant difference in the value of agricultural machinery among households engaged in agricultural production in 2016. However, by 2018, the production capital accumulation in households with existing agricultural machinery was, on average, 1760 yuan higher compared to the "broad-based subsidy" phase, significant at the 5% level. This indicates that the targeted subsidy policy can effectively increase production capital. By 2020, households that previously did not have machinery saw a significant increase in machinery value, amounting to 3330 yuan, significant at the 1% level. This reflects the enduring support of continuous subsidies on capital accumulation in impoverished households, thereby validating Hypothesis 2. The article concludes that increasing government subsidies during the implementation of targeted poverty alleviation and rural revitalization policies effectively improves the economic status of rural households and increases the value of household agricultural machinery, with this positive effect becoming more pronounced over time.

It should be noted that the government subsidy policy impacts the value of agricultural machinery in impoverished households through two pathways: (1) Helping households that already own machinery acquire more or higher-value machinery [25]. (2) Encouraging households without agricultural machinery to purchase it, thereby increasing the proportion of households with agricultural machinery. The former is validated in the sample group with existing agricultural machinery, and the latter through the Probit model, which estimates whether a household owns agricultural machinery as the dependent variable. As shown in the third column of the estimation results, the ownership rate of agricultural machinery in rural households in China significantly increased in 2016 but slightly decreased in 2018. Despite this decrease, the subsidy still had positive effects, with ownership rates in subsidized households being higher by 26.9%, 18.4%, 16.1%, and 17.7% respectively compared with non-subsidized households. Thus, the government subsidy policy can establish a long-term mechanism for narrowing the ownership rate gap between the two groups, particularly effective during the comprehensive promotion of rural revitalization.

A comparison reveals that while the positive effect of government subsidies on the value of household agricultural machinery continuously increased from 2016 to 2020, the impact on ownership rates slightly decreased in 2018 and then rose again in 2020. However, the promotion coefficient in 2020 was lower than in 2016. This phenomenon could be attributed to two reasons: (1) Recently, the proportion of higher-value machinery such as large and medium-sized tractors and combines, owned by farmers has increased, raising the overall value of household machinery. (2) The influence of agricultural machinery cooperatives. Farmers receive government subsidies for machinery purchases through cooperatives and other government support funds. Although the machinery belongs to the cooperative, members benefit from specialist guidance in machine operation and resource management, thereby increasing their income. In fact, there are numerous similar cooperatives in China, and their continuous promotion is widespread.

Additionally, considering that the value of simple tools in the CFPS survey is within 1000 yuan while the value of machinery such as tractors and hullers is significantly higher, the article re-estimates the Probit model using a threshold of agricultural machinery value greater than 1000 yuan to test the robustness of the results. The main conclusions remain consistent with the previous findings and are therefore omitted due to space constraints.

In conclusion, policymakers should continue to enhance government subsidy policies and develop more targeted measures tailored to local circumstances. Additionally, support for the agricultural machinery market and technological advancements should be strengthened to promote continuous and stable growth of the rural economy and advance comprehensive rural revitalization. Through robust policy support and guidance, assisting farmers in establishing and consolidating production capital will not help narrow the regional gap in machinery ownership rates but also lay a solid foundation for the overall improvement of agricultural productivity and rural economic development.

## 6. Conclusion and recommendations

Utilizing the data from China Family Panel Studies (CFPS) and applying a difference-in-differences model that accounts for individual and time fixed effects and control variables, this study finds that government subsidy policies significantly promote per capita net income and the accumulation of agricultural production capital in rural households. Additionally, counterfactual tests confirm the robustness of these conclusions. Given the crucial role of agricultural machinery in future rural household income, this study also examines on the impact of government subsidy policies on the ownership rate of agricultural machinery to assess policy sustainability and provides the following recommendations.

### 6.1 Strengthen government subsidy efforts

As agricultural machinery is the primary tool for agricultural production, effectively utilizing government subsidies for agricultural production is crucial to increasing household per capita net income. For households already owning agricultural machinery, subsequent income improvements from government subsidies are not significantly different from the pre-2014 "broad-based" subsidies, whereas households without agricultural machinery experienced sustained income increases. Therefore, China should continue to increase government subsidies, expand the scale and scope of subsidies, improve the effectiveness of subsidies, consolidate the results of subsidies, and promote comprehensive and rapid development of agricultural mechanization in China.

### 6.2 Establish an incentive-based dynamic government subsidy mechanism

The results of this study indicate that government subsidies have different long-term effects on different types of rural households. Considering that government subsidies are significant fiscal expenditures, their benefits should be fully realized. For households that have been receiving government subsidies for a long period and already possess agricultural machinery, an incentive-based subsidy withdrawal system could be gradually implemented. This would allocate resources to more deserving households while avoiding the phenomenon of welfare dependence, laying the foundation for addressing relative poverty issues. Additionally, in promoting the accumulation of production capital in other rural households, a conditional cash transfer approach commonly adopted in developed countries could be considered. This approach requires recipients of government subsidies to fulfill certain obligations after receiving the subsidy, such as ensuring their children attend school or maintaining employment status, thereby transitioning from "blood transfusions" to "hematopoiesis".

### 6.3 Promote agricultural mechanization and increase machinery ownership rate

Agricultural machinery is the most efficient asset form for household production. Agricultural mechanization is crucial for mitigating natural disasters, freeing up farm productivity, achieving agricultural bumper harvests, enhancing work efficiency, and increasing farmers’ incomes. The government should continue to vigorously promote agricultural mechanization, leveraging the "visible hand" to create favorable conditions for agricultural and rural modernization. Current data show the significant role of government subsidies in enhancing the value of household machinery, through their effect on increasing the ownership rate of agricultural machinery is less pronounced. Although this study verified causality and policy sustainability, the statistical results indicate that even though the 2020 machinery ownership rate of subsidized households increased from 2018, it remained below the 2014 level. This suggests that, despite the elimination of absolute poverty in China, the causes of relative poverty are more complex, highlighting the need to gradually shift the focus of assistance to establishing long-term mechanisms. Future considerations should include enhancing the agricultural machinery ownership rate of rural households, particularly those receiving government subsidies.

### 6.4 Comprehensive improvement of household production capacity

Government subsidies are not just regular livelihood aids; simply increasing fiscal funds without targeted strategies will struggle to maintain sustained effects and fully utilize fiscal resources. The core purpose of subsidies is to enhance household production capacity. This study mainly focuses on agricultural machinery as a common form of physical capital, but in the long term, investments in children’s education and human capital accumulation are equally crucial. Transforming knowledge into productivity and improving production relations are the most effective means of achieving sustainable poverty alleviation. Therefore, practical policy operations should balance short-term and long-term considerations. It is essential to help households accumulate important production tools in the short term and encourage investment in children’s education, strengthen ideological education, cultivate a proactive attitude, and promote independence. Encouraging young people to build their hometowns can transform a significant rural population into human resources that support comprehensive rural revitalization.

## 7. Future research directions

### 7.1 Explore the implementation effectiveness of policies in different regions

From the perspective of China, on the one hand, this study uses 2014 as the implementation year of the subsidy policy during its broad rollout phase, potentially overlooking the policy effects during pilot and expanded phases. Future research could employ multi-period difference-in-differences models for more in-depth analysis. Additionally, the Chinese government should make use of the amplification effects of fiscal funds and their demonstrative role to attract more social capital into the field of agricultural mechanization. On the other hand, future scholars can further compare the effects of government subsidy policies in different provinces or regions of China, explore the impact of regional differences on household income and the value of agricultural machinery, and study the similarities and differences in regional economies, resource endowments, and policy environments to provide a basis for regional policy adjustments.

From an international perspective, future research can compare China’s agricultural subsidy policies with those of other developing countries, exploring the effectiveness of policy implementation and their impact on household income and agricultural mechanization. For example, India implements a wide range of diversified agricultural subsidy policies, including subsidies for fertilizers, seeds, agricultural machinery, and electricity to reduce agricultural production costs and increase farmer income. China’s agricultural machinery purchase subsidy policy focuses more on promoting the level of agricultural mechanization but also emphasizes support for small-scale farmers. Therefore, future analysis can compare and analyze the multidimensional subsidy models in China and India in terms of promoting increased income for small-scale farmers. Brazil focuses on improving agricultural modernization through technological and market support, while China’s subsidies currently focus more directly on agricultural machinery purchases. Although China’s agricultural machinery purchase subsidy policy has increased the level of mechanization, it currently does not provide extensive technical training support. China can learn from Brazil’s approach by strengthening training and technical guidance for Chinese farmers to maximize the efficiency of mechanized equipment use.

Meanwhile, comparisons and analyses can be made with the agricultural subsidy policies of developed countries to provide reference for policy design in developing countries. The European Union’s Common Agricultural Policy has clear advantages in environmental protection and sustainable development, while current research in China focuses more on increasing productivity and farmers’ income. Therefore, China can draw on the EU’s environmental subsidies and sustainable development concepts within the existing subsidy framework to promote green agriculture and policy optimization for sustainable development. Australia provides market support and export subsidies to help farmers expand market channels and enhance the competitiveness of agricultural products, while China’s subsidies mainly target domestic production. Future considerations can include how to introduce market and export support strategies to expand the international market for Chinese agricultural products and increase income sources for farmers. In addition, China’s current policies focus on increasing productivity, and in the future, it can draw on Canada’s experience with income stabilization plans to provide more comprehensive support for farmers.

### 7.2 In-depth investigation of multifaceted subsidy impacts

The government subsidies reported by the CFPS database each have specific policy orientations. The aggregated data from the CFPS used in this study did not allow for a detailed description of the mechanistic effects and impacts of each subsidy on rural household income growth. In the future, further research could compare the effects of direct income subsidies (such as cash transfers) and production capital subsidies (such as agricultural machinery purchase subsidies) on increasing household income and productivity, differentiate between ecological subsidies (such as land reforestation and environmental protection agricultural subsidies) and production subsidies on the overall impact on household income, and explore a balanced approach between ecological protection and increasing household income.

The input subjects of subsidy funds include the government, farm households and enterprises and other social organizations. Due to the limited data related to enterprises and other social organizations, this paper analyzes the productive capital accumulation behavior of farm households and the benefits of government subsidies from both theoretical and empirical perspectives, mainly from the perspectives of the government and farm households. However, the impact of subsidies provided by enterprises and other social organizations on agricultural machinery holdings of farmers’ households still needs further research.

### 7.3 Investigate the influence of new agricultural machinery cooperatives

The focus of this study is on agricultural machinery in rural households in China. However, currently in rural areas of China, there is a growing trend of new agricultural machinery cooperatives, and the agricultural machinery within these cooperatives is considered public assets. It is important to note that other developing countries also exhibit similar trends. For example, Ethiopia has strengthened the market competitiveness and collective bargaining power of farmers by supporting farmer cooperatives and cluster agriculture. Kenya has implemented an agricultural technology and equipment sharing program, established agricultural service centers, and provided agricultural machinery leasing and sharing services, offering farmers a more flexible way to use machinery and alleviating their financial pressures. In the future, China could consider promoting agricultural machinery leasing and sharing services on the basis of agricultural machinery purchase subsidies, further reducing the cost of machinery for farmers and enhancing resource utilization.

Although China’s agricultural subsidy policies primarily target individual farmers, a commonality with other countries is that these new institutions such as agricultural machinery cooperatives utilize various agricultural machinery resources through collective and intensive management, thereby improving machinery efficiency and playing an increasingly important role in promoting agricultural mechanization and enhancing farm productivity. Therefore, future research should further examine the impact of these new institutions on farmer behavior and decision-making, analyzing whether these differences could lead to significant income disparities.
